# A Model Stacking Framework for Identifying DNA Binding Proteins by Orchestrating Multi-View Features and Classifiers

**DOI:** 10.3390/genes9080394

**Published:** 2018-08-01

**Authors:** Xiu-Juan Liu, Xiu-Jun Gong, Hua Yu, Jia-Hui Xu

**Affiliations:** 1 School of Computer Science and Technology, Tianjin University, Nankai, Tianjin 300072, China; 18202578958@163.com (X.-J.L.); yuhua@tju.edu.cn (H.Y.); 2 Tianjin Key Laboratory of Cognitive Computing and Application, Nankai, Tianjin 300072, China; 3 Beijing KEDONG Electric Power Control System Co. LTD, Qinghe, Beijing 100192, China; xujiahui@sgepri.sgcc.com.cn

**Keywords:** DNA-binding proteins, model stacking, logistic regression, multi-view features

## Abstract

Nowadays, various machine learning-based approaches using sequence information alone have been proposed for identifying DNA-binding proteins, which are crucial to many cellular processes, such as DNA replication, DNA repair and DNA modification. Among these methods, building a meaningful feature representation of the sequences and choosing an appropriate classifier are the most trivial tasks. Disclosing the significances and contributions of different feature spaces and classifiers to the final prediction is of the utmost importance, not only for the prediction performances, but also the practical clues of biological experiment designs. In this study, we propose a model stacking framework by orchestrating multi-view features and classifiers (MSFBinder) to investigate how to integrate and evaluate loosely-coupled models for predicting DNA-binding proteins. The framework integrates multi-view features including Local_DPP, 188D, Position-Specific Scoring Matrix (PSSM)_DWT and autocross-covariance of secondary structures(AC_Struc), which were extracted based on evolutionary information, sequence composition, physiochemical properties and predicted structural information, respectively. These features are fed into various loosely-coupled classifiers such as SVM and random forest. Then, a logistic regression model was applied to evaluate the contributions of these individual classifiers and to make the final prediction. When performing on the training dataset PDB1075, the proposed method achieves an accuracy of 83.53%. On the independent dataset PDB186, the method achieves an accuracy of 81.72%, which outperforms many existing methods. These results suggest that the framework is able to orchestrate various predicted models flexibly with good performances.

## 1. Introduction

DNA-binding proteins play a vital role in many biological processes, for instance DNA replication, transactional regulation, DNA repair and DNA modification [1]. It is highly desired to develop computational methods to identify these proteins because of the time consumption and high cost in using experimental techniques such as filter binding assays [2] and X-ray crystallography [3]. So far, many computational methods based on machine learning have been proposed to identify these proteins [4]. Building a meaningful feature set and choosing an appropriate classification algorithm are the two most trivial tasks [5].

Building a feature set usually involves two steps: feature extraction and feature transformation. However, there is no clear boundary in using these two terms. To eliminate confusion, we use the first term to denote both cases in this paper. The information extracted includes sequence-based features [6,7] and structure-based features [8].

The sequence-based feature extraction methods make full use of sequence composition, physiochemical properties and evolutionary information. Liu et al. proposed a protein feature vector representation composed of three kinds of features including overall amino acid composition, Pseudo Amino Acid Composition (PseAAC) and physiochemical distance transformation [9]. They further improved the model by incorporating the evolutionary information by using profile-based protein representation [10]. Xu et al. transformed the Position-Specific Scoring Matrix (PSSM) profiles into uniform numeric vectors for presenting protein sequences by the distance transformation scheme [11]. In [12], the protein sequences represented in the form of amino acids or the physical-chemical properties of amino acids were converted into a series of fixed-length vectors by K-mer composition and the auto-cross covariance transformation. Zhang et al. built feature sets by extracting the evolutionary information from the Position-Specific Frequency Matrix (PSFM), including Residue Probing Transformation (RPT), Evolutionary Difference Transformation (EDT), Distance-Bigram Transformation (DBT) and Trigram Transformation (TT) [13].

As for the structure-based features, they often outperform the sequence-based features because they are more discriminating in identifying DNA-binding proteins. However, the structural information of the protein sequence is not always available because the structure of some protein sequences is unknown [14]. Structural information such as motifs and secondary structures are important for identifying DNA-binding proteins. Chowdhury et al. used sequence evolutionary and structural information embedded in the protein sequences as features, in which the structural information was extracted using the SPIDER 2 software [15,16,17].

There are many classification algorithms applied to identify DNA-binding proteins, such as support vector machine [13,17,18,19,20], Gaussian naive Bayes [21] and Random Forests(RF) [22,23]. Many existing methods employ only a single classification algorithm. The single classifier has its own inherent defects [24]. For instance, SVM has high computational complexity, and its performance relies heavily on its parameters. Logistic regression requires huge samples to achieve stable performance [25]. Comparing to the single classifier, ensemble learning tries to train multiple models using base classifiers, then combines them to make the final predictions [26,27]. There are many effective ensemble strategies such as boosting, bagging, stacking and majority voting. Xu et al. [28] employed the unbalanced-AdaBoost to predict DNA-binding proteins using features of residue/dipeptide compositions and distributions. Song et al. [29] adopted 16 base classifier algorithms and applied the weighted voting to identifying DNA-binding proteins using 188Dfeatures. Liu et al. [27] used the SVM as the base classifier and applied weighted voting to make the final prediction, in which the weights were gained by the grid search.

Having inherent parallelization and a flexible combination and mathematical evaluation of base models, stacking is often used in computationally intensive scenarios with the particular intention to investigate the contributions of base models. Zou et al. applied a stacking-based method for identifying DNA-binding proteins using four types of feature vectors covering global, non-local and local protein sequence information, in which SVM was adopted as both the base and combination model [30].

In this work, we propose a model stacking framework by orchestrating multi-view features and classifiers (MSFBinder) to investigate how to integrate and evaluate loosely-coupled models for predicting DNA-binding proteins. The framework integrates multi-view features including Local_DPP [22], 188D [31], PSSM_DWT [32] and AC_struct [33] for secondary structures, which are extracted based on evolutionary information, sequence composition, physiochemical properties and predicted structural information, respectively. These features are fed into various loosely-coupled classifiers such as SVM and random forest. Then, a Logistic Regression (LR) is applied to evaluate the contributions of these individual classifiers and to make the final prediction. Within the framework, we first train SVM classifiers on the four feature spaces independently to show their prediction effectiveness. Then, we build three predictors including both homogeneous (SVM) and heterogeneous (SVM, Random Forest (RF) and Naive Bayes (NB)) base models. After plotting and T-test analysis of LR coefficients on both the training and independent test datasets, we find that RF prefers the features of PSSM_DWT, SVM likes Local_DPP features more and both of these two kinds of features play significant roles in the prediction of DNA-binding proteins. It is noteworthy that the NB classifier is more stable against all the features, although its coefficients are not too high. Meanwhile, all three predictors outperform the single classifiers, and there is no big performance difference between homogeneous and heterogeneous base models. Finally, we show that the overall performance of MSFBinder outperforms the state-of-the-art of existing methods on the independent testing dataset.

## 2. Materials and Methods

**Figure 1:**
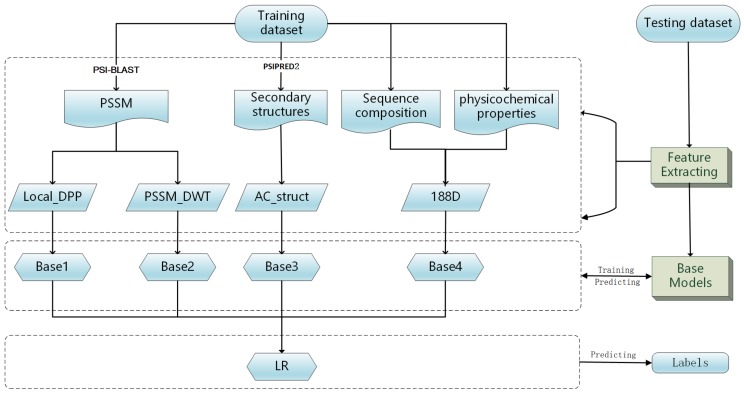
The MSFBinder workflow.

The framework of the presented method is illustrated in Figure 1. It consists of two stages: training and predicting phases. In the training phase, the training samples are transformed into feature vectors by four feature extraction methods. Then, base models are trained on the four feature spaces independently. The predicted probabilities are fed into the LR classifier to make the final prediction. In the prediction phase, the testing samples are transformed into feature vectors using the same feature extraction methods. Then, the models previously trained are used to predict DNA-binding proteins.

### 2.1. Datasets

Two benchmark datasets PDB1075 [18] and PDB186 [21] were used to evaluate the performance of the proposed method. PDB1075 contains 525 DNA-binding proteins and 550 non-DNA-binding proteins, and PDB186 contains 93 DNA-binding proteins and 93 non-DNA-binding proteins. The details of the two datasets are shown in Table 1. The protein sequences in the benchmark datasets were derived from the Protein Data Bank (http://www.rcsb.org/pdb/home/home.do). We used the PDB1075 dataset for training the models and the PDB186 dataset for the independent test. Protein sequences in the benchmark datasets were selected according to the following 3 criteria: (1) the length of protein sequences is not less than 50; (2) the protein sequence cannot contain unknown residues such as X; (3) the sequence similarity between any two proteins is less than 25%.

### 2.2. Feature Extraction

Four kinds of feature extraction methods were used. They were Local_DPP, PSSM_DWT, AC_struct and 188D. Their details are explained below.

#### 2.2.1. Features Based on Evolutionary Information

The sequence evolutionary information can be constructed from the Position-Specific Scoring Matrix (PSSM). It has been applied in many similar works, such as protein fold recognition [34], ATP binding site prediction [6], bacteriophage virion identification [35] and aptamer-protein interacting pair prediction [33]. Local_DPP extracts local evolutionary information from the PSSM matrix by dividing the matrix into *n* parts, and PSSM_DWT gains discriminatory information from the PSSM matrix by compressing the matrix using the Discrete Wavelet Transform (DWT) method.

Suppose that the number of amino acids in a given protein sequence is *L*; the PSSM matrix is defined as:

(1) $$P={\left[\begin{array}{cccc} p_{1,1} & p_{1,2} & \cdots & p_{1,20}\\ \vdots & \vdots & \vdots & \vdots \\ p_{i,1} & p_{i,2} & \cdots & p_{i,20}\\ \begin{array}{c} \vdots \\ p_{L,1} \end{array} & \begin{array}{c} \vdots \\ p_{L,2} \end{array} & \begin{array}{c} \vdots \\ \cdots \end{array} & \begin{array}{c} \vdots \\ p_{L,20} \end{array} \end{array}\right]}_{L\times 20}$$

The rows in the matrix correspond to the positions in the sequence, and the columns correspond to one of the twenty types of amino acids. The values of the *i*-th row in the matrix represent the probabilities of the *i*-th position to be each type of the 20 amino acids. The PSSM matrix is normalized by the following formula.

(2) $$p_{i,j}^{\prime }=\frac{p_{i,j}-p_{j}}{S_{j}}\left(i=1,2,\ldots ,20;j=1,2,\ldots ,L\right)$$

where $p_{i,j}$ is the original value in the PSSM matrix and $p_{j}$ and $S_{j}$ are the mean and standard deviation of the *j*-th row.

PSSM was obtained from the PSI-BLAST [36] program with iterations of 3 and an E-value of 0.001.

##### Local_DPP

Local_DPP [22] extracts local evolutionary features from the PSSM matrix. Firstly, it partitions the PSSM matrix into *n* segments. The length of the first $\left(n-1\right)$ segments is ${}^{L}/{}_{n}$, and the length of the *n*-th segment is $L-(n-1)\times \left({}^{L}/{}_{n}\right)$. The following formulas are applied to extract features for each segment

(3) $${Part}_{1}=\left\{F_{j}\left(k\right)=\frac{1}{length(k)}\sum_{i=0}^{length(k)-1}p_{i,j}^{\prime }j=1,2,\ldots ,20\right\}$$

(4) $${Part}_{2}=\left\{\phi_{j}^{\xi }\left(k\right)=\frac{1}{length(k)-\xi }\sum_{i=0}^{length(k)-1-\xi }{\left(p_{i,j}^{\prime }-p_{i+\xi ,j}^{\prime }\right)}^{2}\xi =1,2,\ldots ,\lambda ,1<\lambda <length\left(K\right)\right\}$$

where *k* represents the *k*-th segment, length(k) represents the length of the *k*-th segment and $p_{i,j}^{\prime }$ represents the normalized value in the PSSM matrix. length(K) denotes the minimum length of the segments. In the *k*-th segment, $F_{j}\left(k\right)$ represents the average probability of occurrence of the *j*-th type of amino acid at each position in the segmented sequence during the evolutionary process. $\phi_{j}^{\xi }\left(k\right)$ denotes the average correlation for the *j*-th type of amino acid between two residues separated by ξ. ${Part}_{1}$ contains the local evolutionary information, and ${Part}_{2}$ contains the sequence order information.

Hence, for each segment, we can obtain 20D features according to ${Part}_{1}$ and 20×λD features according to ${Part}_{2}$. There are in total *n* segments, and we then gain 20×(1+λ)×n D local features.

##### PSSM_DWT

**Figure 2:**
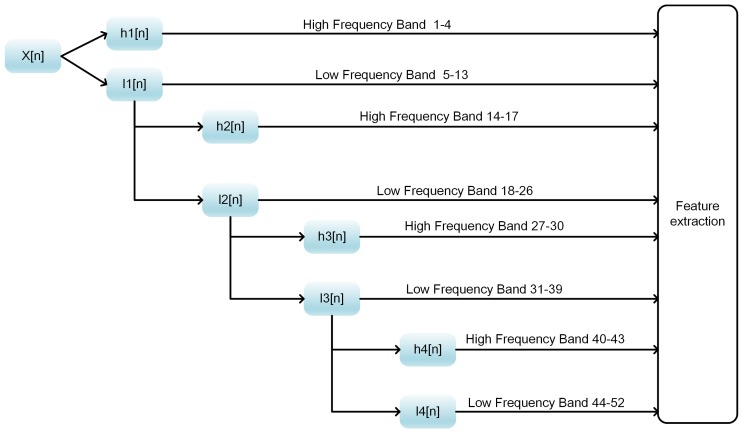
Discrete Wavelet Transform (DWT) feature extraction. $h_{i}\left[n\right]$ denotes the high pass filter, which can filter out the low frequency band of the discrete signal and retain the high frequency band, and $l_{i}\left[n\right]$ denotes the low pass filter.

PSSM_DWT [32] extracts features from the given sequences by DWT [37]. DWT can discretely sample the wavelets and grasp both the frequency and location information [38]. When applied to the given protein sequence, DWT decomposes it into a list of coefficients at different dilations and then remove noise information of the sequence from the profiles. According to Nanni et al. [39,40], DWT can be defined as:

(5) $$y_{j,low}\left[n\right]=\sum_{k=1}^{N}x[k]g[2n-k]$$

(6) $$y_{j,high}\left[n\right]=\sum_{k=1}^{N}x[k]h[2n-k]$$

where *N* denotes the length of the discrete signal, x[n] denotes the discrete signal, *g* denotes the low pass filter and *h* represents the high pass filter. $y_{j,low}\left[n\right]$ represents the approximate coefficient of the low frequency part of the signal, and $y_{j,high}\left[n\right]$ denotes the detailed coefficient of the high frequency part of the signal. With the decomposition process being repeated, the frequency resolution and approximation coefficients can be further increased. In this study, we employed 4 levels of DWTs, and the schematic diagram is shown in Figure 2.

As shown in Figure 2, at each level of DWT, the high-frequency band and the low-frequency band of the discrete signal are separated. The maximum, minimum, average and standard deviation for each band are calculated. For a given amino acid, 13×4=52D features are obtained after DWT transformation. Finally, we gain 52×20=1040D features for a total of 20 types of amino acids.

#### 2.2.2. Features Based on Sequence Composition and Physiochemical Properties

The 188D [31] extracts the sequence features according to the Composition (C), Distribution (D), bivalent Frequency (F) and physiochemical properties of amino acids and uses a 188-dimensional vector to encode the raw sequence. The first 20-dimensional features were obtained by calculating the appearance frequency of every amino acid. Subsequently, the amino acids were divided into three different categories based on the eight physiochemical properties of proteins; see Table 2 [41,42,43,44]. For each protein property, three dimensions were for the occurrence frequencies of the three categories, and three dimensions were for the occurrence frequencies of three bivalent classes in which the two amino acids were from different categories. Dividing the entire protein sequence into five equal parts and calculating the distribution frequencies of each class in the five parts (the first 25%, 50%, 75% and 100%) yielded the next fifteen dimensions. For all eight properties, there were (3+3+15)×8=168 features. Finally, a 168+20=188-dimensional feature vector was used to represent a protein sequence.

#### 2.2.3. Features Based on Predicted Secondary Structures

##### AC_struct

The predicted secondary structural information of protein sequences were derived from PSIPRED2 [45], which is a state-of-the-art algorithm. For each residue in the given sequence, PSIPRED 2 provides the probability profile of three secondary structure states (helix, strand and coil).

For a given sequence with length *L*, the L×3 matrix is defined as:

(7) $$S={\left[\begin{array}{ccc} s_{1,1} & s_{1,2} & s_{1,3}\\ s_{2,1} & s_{2,2} & s_{2,3}\\ \begin{array}{c} \vdots \\ s_{i,1}\\ \vdots \\ s_{L,1} \end{array} & \begin{array}{c} \vdots \\ s_{i,j}\\ \vdots \\ s_{L,2} \end{array} & \begin{array}{c} \vdots \\ s_{i,3}\\ \vdots \\ s_{L,3} \end{array} \end{array}\right]}_{L\times 3}$$

where $s_{i,j}$ denotes the probability that the *i*-th residue belongs to the *j*-th type of the secondary structure.

In order to make the fixed-length vector of protein sequences from the above matrix, we employ the auto-covariance transformation [46].

The Auto Covariances (AC) is defined as:

(8) $${AC}_{\lambda ,j}=\frac{1}{\left(L-\lambda \right)}\sum_{i=1}^{L-\lambda }\left(s_{i,j}-\bar{s_{j}}\right)\times \left(s_{i+\lambda ,j}-\bar{s_{j}}\right),\left(\lambda =1,2,\ldots ,L_{\min}-1\right),\left(j=1,2,3\right)$$

where $\bar{s_{j}}$ denotes the average of the probabilities for the *j*-th type of the secondary structure. λ denotes the distance between any two amino acids in the given sequence. $L_{\min}$ denotes the minimum length of the protein sequences, which is set to 50 in this work.

The auto-covariances within 50 amino acids of a protein sequence produced 49×3=147-dimensional features. The average probabilities and standard deviations of the three types of secondary structures consisted of the next 6-dimensional features. Finally, a total of 153 features were obtained.

The numbers of features via the four extraction methods are summarized in Table 3

### 2.3. Classification Algorithms

#### 2.3.1. Support Vector Machine

Support vector machine [47] constructs a hyper-plane in high-dimensional space or in infinite-dimensional space and can be employed for classification, regression and other tasks. SVM was originally used to solve the two-class problems and later extended to the multiclass problems. For the two-class problems, SVM maps data to a high-dimensional feature space to find the best hyper-plane. In this study, we select the Radial Basis Function (RBF) as the kernel function, since it has been proven to be very effective in many applications such as protein-ATP binding site prediction [6]. The grid search approach was applied to find out the optimal capacity parameter *C* and kernel width *g*.

#### 2.3.2. Random Forest

Random Forest, a type of ensemble machine learning algorithm, performs a majority voting on multiple decision tree models, each of which is independently constructed with a bootstrap sample, on both the feature and instance axises, of the training set. It corrects for decision trees’ habit of overfitting their training set. Because of its outstanding prediction performance, RF has been adopted in many scientific research fields, especially in bioinformatics [48]. RF has numerous parameters to be tuned, such as the number of trees to be built before taking majority voting, the maximum number of features to train a tree and the minimum sample leaf size in an individual tree. Again, the grid search is used to tune these parameters.

#### 2.3.3. Naive Bayes

Naive Bayes is a type of probabilistic classifier based on applying Bayes’ theorem with the strong independence assumption between features. Although this assumption does not hold for most cases, NB has very sound performances in many applications [49,50].

For a given input $X=(x_{1},\ldots ,x_{n})$, NB assigns it a class label *c* by optimizing the posterior:

(9) $$c=arg\max_{c}p\left(cx_{1},\ldots ,x_{n}\right)=arg\max_{c}p\left(c\right)\prod_{i=1}^{n}p\left(x_{i}c\right)$$

In this work, we use the multinomial Gaussian distribution to estimate its parameters.

#### 2.3.4. Model Stacking

In contrast to the ordinary classifier, which tries to learn one hypothesis from training data, ensemble learning tries to construct a set of hypotheses and combine them for use. An ensemble contains a number of learners, which are usually called base learners. The generalization ability of an ensemble is usually much stronger than that of base learners. However, this is not always true since using not-so-weak base learners often results in better performance. Bagging, boosting, stacking and voting are some of typical ensemble strategies [51]. Bagging trains a number of base learners, each using a different bootstrap sample. It combines them by majority voting, and the most-voted class is predicted. Boosting uses subsets of the training set to produce a series of averagely performing models and then “boosts” their performance by combining them together using a particular cost function such as majority vote. The majority voting assigns the final class label of each sample using the one predicted by the (weighted) majority of base classifiers. Stacking utilizes another classifier to combine the results of the base classifiers. Because of the inherent parallelization, flexible combination and mathematical evaluation of base classifiers in the stacking strategy [52], we use it as the base framework to combine base classifiers and to make the final prediction using logistic regression.

Logistic regression is a mathematical method modeling the logistic relationship between categories and numerical feature vectors [53]. It uses the sigmoid function to solve the two-class problems and the softmax function to solve multi-class problems. For the two-class problems, given the protein sequence and the corresponding feature vectors, the relationship between two-class labels and feature vectors $\left(F=f_{1},\ldots ,f_{N}\right)$ is defined as:

(10) $$P=\frac{1}{1+e^{-(\theta_{0}+\theta_{1}f_{1}+\ldots +\theta_{N}f_{N})}}$$

where $\theta_{0}$ to $\theta_{N}$ are the unknown parameters, which can be obtained by maximum likelihood estimation.

### 2.4. Performance Measures

We employed leave-one-out cross-validation and five-fold cross-validation to evaluate the performances of predictor capability. In the five-fold cross-validation, the training dataset was randomly divided into five equal parts, one for the testing dataset and the others for training datasets. As for the leave-one-out cross-validation, each sample in the training dataset was used for testing, and the remaining samples were used for training.

Four evaluation metrics, Sensitivity (SN), Specificity (SP), Accuracy (ACC) and Matthew’s Correlation Coefficient (MCC), were used as the performance indicators. Their formulas are as follows:

(11) $$SN=\frac{TP}{TP+FN}$$

(12) $$SP=\frac{TN}{TN+TP}$$

(13) $$ACC=\frac{TP+TN}{TP+FP+TN+FN}$$

(14) $$MCC=\frac{TP\times TN-FP\times FN}{\sqrt{\left(TP+FN\right)\left(TP+FP\right)\left(TN+FP\right)\left(TN+FN\right)}}$$

where TP (TN) denotes the number of positive (negative) samples correctly predicted and FP (FN) denotes the number of positive (negative) samples incorrectly predicted.

All the source codes and data are available on the GitHub server https://github.com/gongxjtju/MSFBinder.

## 3. Results and Discussions

### 3.1. Performance Comparisons of Different Feature Representations Only Using SVM as the Training Model

In order to evaluate the prediction capability of the different feature extraction methods, the five-fold cross-validation was applied using SVM as the training model. As shown in Table 4, most of the feature spaces achieved acceptable performance. Local_DPP beat all the other three with an accuracy of 78.32%, an MCC of 0.5681 and an SP of 75.63%. The results suggest that sequence order and the local evolutionary information contribute significantly to the prediction of DNA-binding proteins. The performance of AC_struct was inferior to the others. This might have been caused by the false predictions of the secondary structures by the PSIPRED 2 program.

### 3.2. Performance Comparisons Using Different Base Classifiers

In this section, we build three types of predictors: MSFBinder (SVM), MSFBinder (SVM, RF) and MSFBinder (SVM, RF, NB) in the MSFBinder framework. They were performed on four types of feature spaces using SVM, SVM + RF and SVM + RF + NB as the base classifiers and obtained 4, 8 and 12 models, respectively. Then these models are fed into the LR to make the final predictions. For each predictor, we compared their performances to two types of parameter setups of Local_DPP. The leave-one-out cross-validation was applied to evaluate the performances. The results on PDB1075 and PDB186 are shown in Table 5 and Table 6.

Table 5 shows that the second predictor with n=3 and λ=1 achieved the best performance with ACC=84.84%, MCC=0.6969, SN=85.52% and SP=84.18%. The first predictor with n=3 and λ=1 performed the worst with ACC=83.35%, MCC=0.6670, SN=83.62% and SP=83.09%. The gaps of ACC, MCC, SN and SP between the best and worst ones were 1.49%, 0.0299, 1.9% and 1.09%, respectively. For the test dataset shown in Table 6, the first predictor with n=2 and λ=2 achieved the best performance. The second predictor with n=3 and λ=1 reached the worst case. The best one beat the worst one in terms of values of ACC, MCC, SN and SP by 2.69%, 0.0389, −3.22 and 8.6%, respectively. Although the first predictor performed slight worse on the training dataset, it worked best on the testing dataset. This suggests that the first predictor had good generalization. All three predictors outperformed the one only using SVM.

### 3.3. Significance Analysis of Different Base Models for Three Predictors

**Figure 3:**
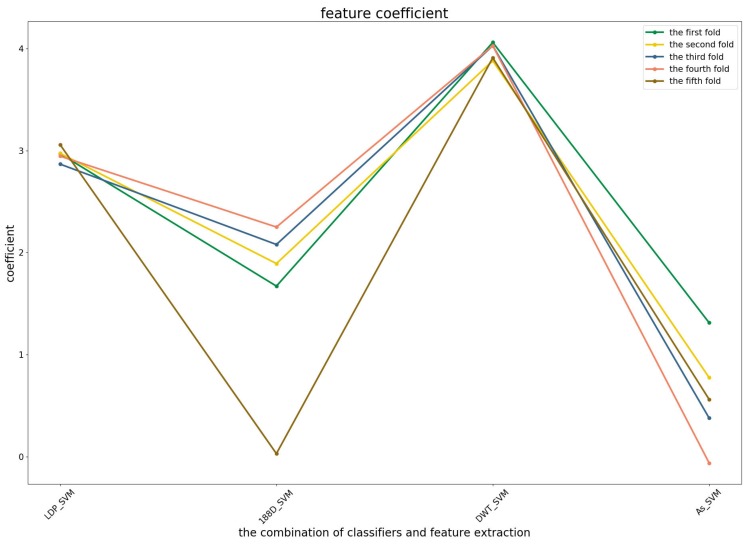
The coefficients of the four base models in the first predictor.

**Figure 4:**
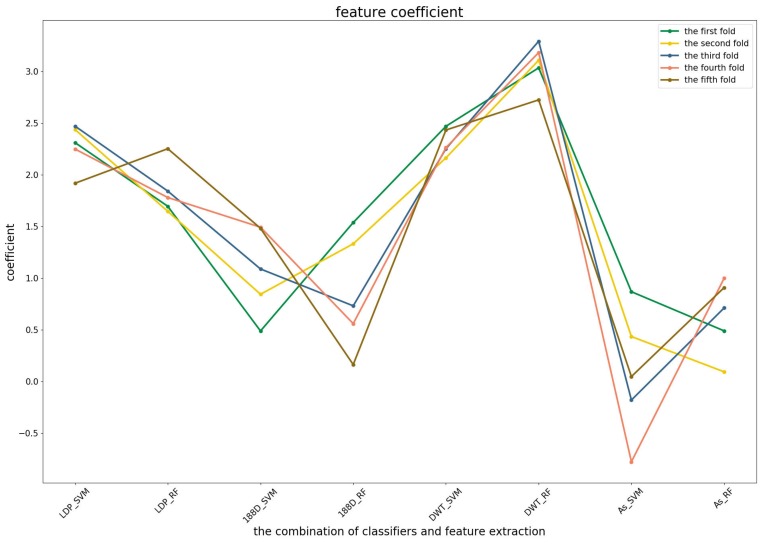
The coefficients of the eight base models in the second predictor.

**Figure 5:**
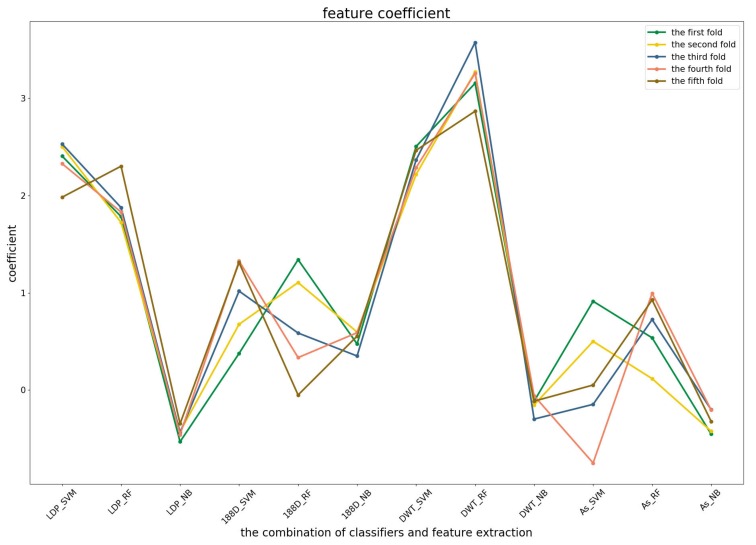
The coefficients of the twelve base models in the third predictor.

To further demonstrate the contributions of different base classifiers combined with corresponding feature representations for the final prediction, the LR coefficients of the base models for five-fold cross-validations are drawn in Figure 3, Figure 4 and Figure 5, in which all the parameters of Local_DPP are set the same, as n=2 and λ=2.

In the first predictor (Figure 3), the PSSM_DWT features contributed the greatest, then the Local_DPP features. Both features were more stable among all of the five-fold cross-validations. The other two features were heavily dependent on the datasets.

Figure 4 shows that the RF model preferred the features of PSSM_DWT, and their combination made the biggest contribution across all of the five-fold cross-validations. SVM combined with the Local_DPP features ranked second. Both two types of features worked well on the SVM and RF classifiers. The 188D features were not sensitive to the datasets for SVM and RF, while the contribution of AC_struct features was smaller than the others for all folds.

For the third predictor (Figure 5), Local_DPP and PSSM_DWT features played similar roles as the second one for SVM and RF. Both of them had significant effects on the predictions. The 1880D features were sensitive to the datasets for RF and SVM, and a similar case applied to the AC_struct and SVM models. It should be noted that NB classifiers were more stable across all of the features, but have a lower contribution.

### 3.4. T-Test Analysis of Different Base Models for Three Predictors and Performance Measures

**Figure 6:**
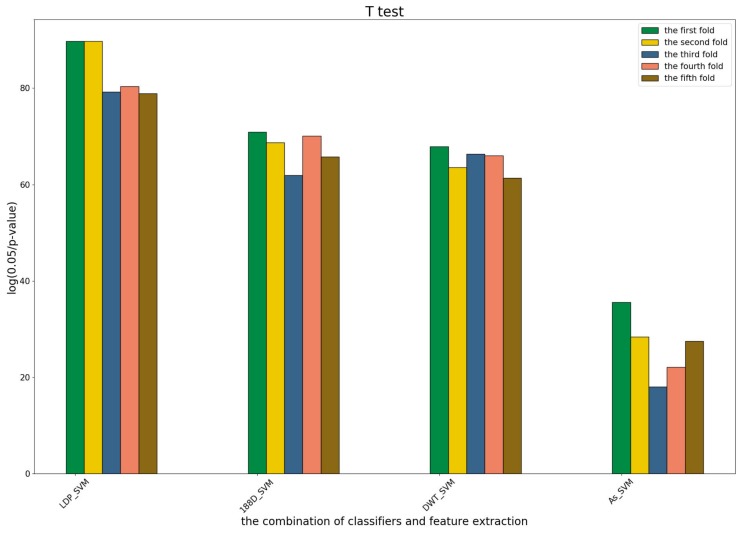
T-test for the first predictor. The meaning of the y-axis denotes the distance between the *p*-value and the threshold. The larger the value of the *y*-axis, the greater the distance. The *x*-axis denotes the combination of different classifiers and feature extraction methods.

**Figure 7:**
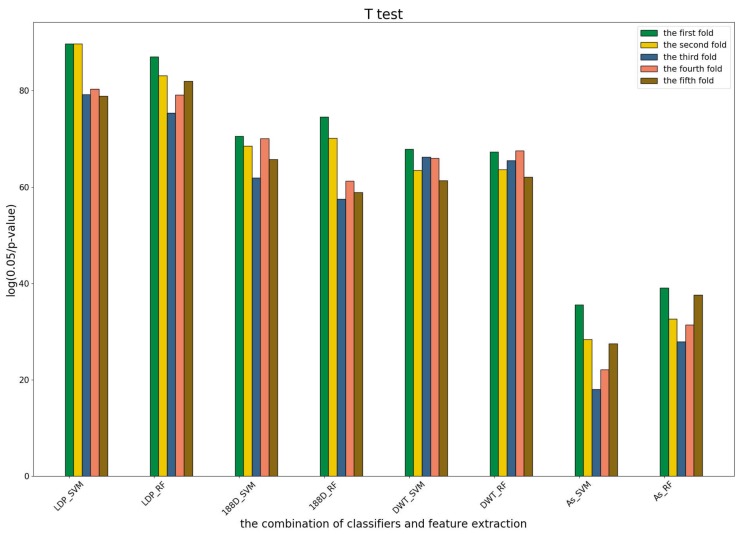
T-test for the second predictor.

**Figure 8:**
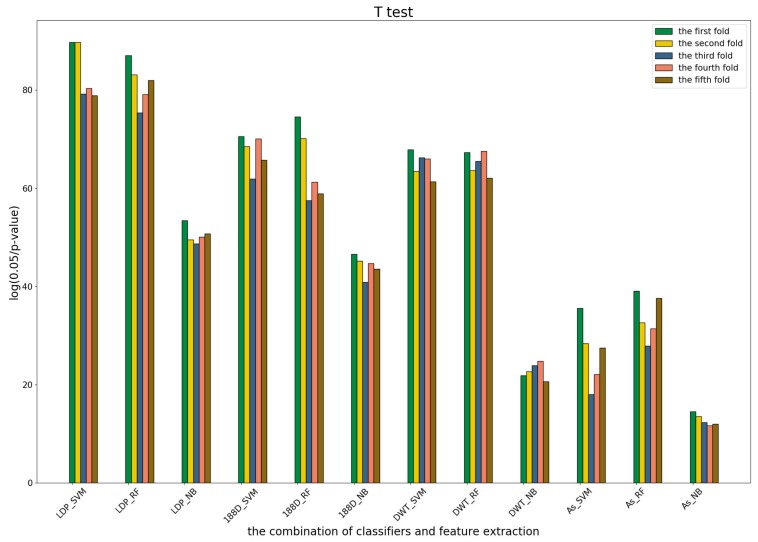
T-test for the third predictor.

To further show the statistical significance of base models for the three predictors, we performed T-test analysis on the LR base models of each predictor for the five-fold cross-validations. The results are shown in Figure 6, Figure 7 and Figure 8.

These results demonstrate that there was no big difference in the *p*-values for all the base models across all folds, except for the ones involved in the AC_struct features. SVM performed the best with all feature spaces.

### 3.5. Performance Comparisons with Single Classifiers

To verify the effectiveness of the presented method, we compared the performances to the ones only using the single classifiers by leave-one-out cross-validation. Three classifiers, SVM, RF and LR, were used. The results are shown in Table 7. MSFBinder with n=2,λ=2 achieved better performance than any of the others, and MSFBinder with n=3,λ=1 performed slight worse than the first one. Among these single classifiers, the SVM model with n=2,λ=2 works the best with ACC=82.60%, MCC=0.6527, SN=84.19% and SP=81.09%. Though the SN value of MSFBinder was lower than the SVM model, its values of AC, MCC and the SP were higher than those of the other single classifiers.

### 3.6. Performance Comparisons with Majority Voting-Based Methods

The majority voting strategy is another popular method of model ensembling. It assigns the final class label of each sample using the one predicted by the (weighted) majority of base classifiers. We ran SVM, RF and LR on the four feature spaces, respectively, then the simple majority voting was applied to make the final predictions; see Table 8 for the results. The ACC, MCC and SN values of MSFBinder were 1.93%, 3.83% and 1.73% higher than the best ones of majority voting-based methods, respectively. The MSFBinders with two types of parameters were both better than the majority voting methods. Compared to Table 4, both kinds of model ensembling performed better than the models only using single classifiers.

### 3.7. Stability Comparisons with the Single Classifiers and Majority Voting Methods

The stability of a learning algorithm refers to the changes in the output of the system when we change the training dataset. A learning algorithm is said to be stable if the learned model does not change much when the training dataset is modified. There are several ways o modify the training set, such as choosing different subsets, using different feature sets and putting noise into the training set. Mathematically speaking, there are many ways of determining the stability of a learning algorithm. Some of the common methods include hypothesis stability, error stability and leave-one-out cross-validation stability.

Here, we tested the stability using different subsets by 5 × 5-fold cross-validation methods. By running the five-fold cross-validation five times on the training set using the four feature sets, the means and standard deviations of four evaluation metrics were calculated; see Table 9.

The results show that MSFBinder achieved the best mean performance with acc=83.70%, MCC=0.6744, SN=84.47% and SP=83.19%. It beat RF in terms of the values ACC, MCC, SN and SP by 2.34%, 0.0474, 3.37% and 1.59%, respectively. While RF beat the minimal standard deviations for all the measures, this suggests that RF was the most stable of them. The standard deviations for the above four measures in MSFBinder ranked 5, 5, 2 and 4 among seven methods, respectively.

### 3.8. Comparisons to Existing Methods

We compared the performances to other existing methods including IDNA-Protdis [18], IDNA-Prot [19], DNA-Prot [54], DNAbinder [20], Kmer1 + ACC [12], Local-DPP [22], PSSM_DT [9], PSSM-DBT [13], iDNAPro-PseAAC [10] and iDNAProt-ES [17]. The results are shown in Table 10 for the training set and Table 11 for the testing set.

On the training set, iDNAProt-ES ranked first for all the evaluation metrics, and MSFBinder ranked second except for the SP metrics. The best performance of iDNAProt-ES might lie in its feature set incorporating various transformations for both the sequence evolutionary information and predicted secondary structure information, while our method only used the auto-covariance features for predicted secondary structure information.

On the testing set, MSFBinder (*n* = 2, λ = 2) ranked the first in terms of ACC (81.72%), and its MCC (0.6417) and SN (93.55%) values were almost the same as the best ones (0.647 and 94.62%) of the others.

It is noteworthy that the values of its ACC, SN and SP were 6.65%, 6.57% and 6.73% higher than ours on the training dataset, while the values of ACC, MCC and SN in our method were 1.08%, 0.0287 and 7.94% higher than iDNAProt-ES. Thus, MSFBinder has better generalization ability than existing methods.

## 4. Conclusions

Orchestrating features and classification algorithms is one of the most tedious works in predicting spatial structures or functions of biological sequences. The stacking model provides a way to combine and to evaluate loosely-coupled base models. In this paper, we propose a model stacking framework by integrating multi-view features and classifiers to investigate how to combine and validate these base models for predicting DNA binding proteins. Integrative experiments demonstrated that MSFBinder has a state-of-the-art performance on both the training and testing datasets and outperforms most of the existing methods. We also explored some characteristics of base classifiers matching corresponding feature spaces. For instance, RF prefers the features of PSSM_DWT; SVM likes the features of Local_DPP more; and both of these two features play significant roles in the prediction of DNA-binding proteins. It is noteworthy that the NB classifier is more stable against all the features, although its coefficients are not too high. Meanwhile, all three predictors outperform the single classifiers, and there is no big performance difference between homogeneous and heterogeneous base models. These findings might yield important clues for designing new feature extraction and classification algorithms for future proteomics studies.

## Figures and Tables

**Table 1:** The two benchmark datasets PDB1075 and PDB186.

	DNA-Binding Proteins	Non-DNA-Binding Proteins	The Ratio	
PDB1075	525	550	0.9545	
PDB186	93	93	1	

**Table 2:** Categories of the eight physiochemical properties.

Physiochemical Property	the 1st Class	the 2nd Class	the 3rd Class
hydrophobicity	RKEDQN	GASTPHY	CVLIMFW
normalized Van der Waals volume	GASCTPD	NVEQIL	MHKFRYW
polarity	LIFWCMVY	PATGS	HQRKNED
polarizability	GASDT	CPNVEQIL	KMHFRYW
charge	KR	ANCQGHILMFPSTWYV	DE
Surface tension	GQDNAHR	KTSEC	ILMFPWYV
Secondary structure	EALMQKRH	VIYCWFT	GNPSD
Solvent accessibility	ALFCGIVW	RKQEND	MPSTHY

**Table 3:** Number of features via different extraction methods.

Local_DPP	PSSM_DWT	188D	AC_struct
20(1+λ)n	1040	188	153

**Table 4:** The performances of different feature representations.

	ACC (%)	MCC	SN (%)	SP (%)
Local_DPP (n=2, λ = 2)	78.32	0.5681	81.14	75.63
Local_DPP (n=3, λ = 1)	77.30	0.5539	84.57	70.36
188D	75.16	0.5034	75.81	74.55
PSSM_DWT	72.47	0.4488	70.67	74.18
AC_struct	68.00	0.3595	63.62	72.18

**Table 5:** The performances of three predictors on PDB1075.

	ACC (%)	MCC	SN (%)	SP (%)
MSFBinder (SVM) (*n* = 2, λ = 2)	83.53	0.6707	83.81	83.27
MSFBinder (SVM) (*n* = 3, λ = 1)	83.35	0.6670	83.62	83.09
MSFBinder (SVM, RF) (*n* = 2, λ = 2)	84.74	0.6948	84.95	84.55
MSFBinder (SVM, RF) (*n* = 3, λ = 1)	84.84	0.6969	85.52	84.18
MSFBinder (SVM, RF, NB) (*n* = 2, λ = 2)	84.65	0.6935	86.10	83.27
MSFBinder (SVM, RF, NB) (*n* = 3, λ = 1)	84.28	0.6859	85.33	83.27

**Table 6:** The performances of the three predictors on PDB186.

	ACC (%)	MCC	SN (%)	SP (%)
MSFBinder (SVM) (*n* = 2, λ = 2)	81.72	0.6417	89.25	74.19
MSFBinder (SVM) (*n* = 3, λ = 1)	79.57	0.6160	93.55	65.59
MSFBinder (SVM, RF) (*n* = 2, λ = 2)	81.18	0.6343	90.32	72.04
MSFBinder (SVM, RF) (*n* = 3, λ = 1)	79.03	0.6028	92.47	65.59
MSFBinder (SVM, RF, NB) (*n* = 2, λ = 2)	80.65	0.6276	91.40	69.89
MSFBinder (SVM, RF, NB) (*n* = 3, λ = 1)	80.11	0.6215	92.47	67.74

**Table 7:** Performance comparisons with single classifiers.

	ACC (%)	MCC	SN (%)	SP (%)
SVM (*n* = 2, λ = 2)	82.60	0.6527	84.19	81.09
SVM (*n* = 3, λ = 1)	82.14	0.6434	83.62	80.73
RF (*n* = 2, λ = 2)	81.58	0.6315	81.33	81.82
RF (*n* = 3, λ = 1)	80.84	0.6166	80.76	80.91
LR (*n* = 2, λ = 2)	81.86	0.6371	81.71	82.00
LR (*n* = 3, λ = 1)	82.33	0.6465	82.67	82.00
MSFBinder (SVM) (*n* = 2, λ = 2)	83.53	0.6707	83.81	83.27
MSFBinder (SVM) (*n* = 3, λ = 1)	83.35	0.6670	83.62	83.09

**Table 8:** The performance comparisons to the majority voting-based methods.

	ACC (%)	MCC	SN (%)	SP (%)
Majority voting (LR) (*n* = 2, λ = 2)	81.08	0.6230	78.89	83.24
Majority voting (LR) (*n* = 3, λ = 1)	81.39	0.6296	79.55	83.33
Majority voting (RF) (*n* = 2, λ = 2)	81.60	0.6338	82.51	80.94
Majority voting (RF) (*n* = 3, λ = 1)	81.28	0.6252	81.71	80.98
Majority voting (SVM) (*n* = 2, λ = 2)	81.77	0.6361	82.28	81.39
Majority voting (SVM) (*n* = 3, λ = 1)	81.08	0.6234	82.74	79.63
MSFBinder (SVM) (*n* = 2, λ = 2)	83.70	0.6744	84.47	83.19
MSFBinder (SVM) (*n* = 3, λ = 1)	82.47	0.6503	82.96	82.08

**Table 9:** The means and standard deviations for the 5 × 5-fold cross-validations.

	ACC	MCC	SN	SP
LR	81.19±0.003265	0.6237±0.006542	80.76±0.0095	81.60±0.006984
RF	81.36±0.001808	0.6270±0.003686	81.10±0.004119	81.60±0.002393
SVM	82.06±0.005233	0.6415±0.01039	82.86±0.007984	81.31±0.01032
Majority voting (LR)	81.08±0.004448	0.6230±0.009077	78.89±0.010404	83.24±0.004515
Majority voting (RF)	81.60±0.006189	0.6338±0.01268	82.51±0.007972	80.94±0.009137
Majority voting (SVM)	81.77±0.006162	0.6361±0.01267	82.28±0.007345	81.39±0.009891
MSFBinder (SVM)	83.70±0.005663	0.6744±0.01116	84.47±0.004215	83.19±0.004991

**Table 10:** Performance comparisons to existing methods on the training set.

	ACC (%)	MCC	SN (%)	SP (%)
IDNA-Protdis	77.30	0.54	79.40	75.27
IDNA-Prot	75.40	0.50	83.81	64.73
DNA-Prot	72.55	0.44	82.67	59.76
DNAbinder (dimension = 400)	73.58	0.47	66.47	80.36
DNAbinder (dimension = 21)	73.95	0.48	68.57	79.09
iDNAPro-PseAAC	76.56	0.53	75.62	77.45
Kmer1 + ACC	75.23	0.50	76.76	73.76
Local-DPP (*n* = 3, λ = 1)	79.10	0.59	84.80	73.60
Local-DPP (*n* = 2, λ = 2)	79.20	0.59	84.00	74.50
PSSM_DT	79.96	0.62	78.00	81.91
PSSM-DBT	81.02	0.62	84.19	78.00
iDNAProt-ES	**90.18**	**0.8036**	**90.38**	**90.00**
MSFBinder (SVM) (*n* = 2, λ = 2)	83.53	0.67	83.81	83.27
MSFBinder (SVM) (*n* = 3, λ = 1)	83.35	0.67	83.62	83.09

**Table 11:** Performance comparisons to existing methods on the testing dataset.

	ACC (%)	MCC	SN (%)	SP (%)
IDNA-Protdis	72.0	0.445	79.5	64.5
IDNA-Prot	67.2	0.344	67.7	66.7
DNA-Prot	61.8	0.240	69.9	53.8
DNAbinder	60.8	0.216	57.0	64.5
iDNAPro-PseAAC-EL	71.5	0.442	82.8	60.2
iDNA-KACC-EL	79.0	0.611	**94.62**	63.4
Kmer1 + ACC	71.0	0.431	82.8	59.1
Local-DPP (*n* = 3, λ = 1)	79.0	0.625	92.5	65.6
Local-DPP (*n* = 2, λ = 2)	77.4	0.568	90.3	64.5
PSSM_DT	80.00	**0.647**	87.09	72.83
PSSM-DBT	80.65	0.624	90.32	70.97
iDNAProt-ES	80.64	0.6130	81.31	**80.00**
MSFBinder (SVM) (*n* = 2, λ = 2)	**81.72**	0.6417	89.25	74.19
MSFBinder (SVM) (*n* = 3, λ= 1)	79.57	0.6160	93.55	65.59
